# Extirpation of Goître

**Published:** 1851-03

**Authors:** 


*Extirpation of Goître.*—The French Académie de Médicine, has lately been occupied with an interesting discussion on the *extirpation of goitre.* The debate originated in a case narrated by M. Roux, of a young man who was the subject of a tumor over the thyroid cartilage the size of a fist. The tumor was very hard and lobulated, and painless, and although it adhered closely to the larynx, it did not materially intefere with respiration. The patient wished to get rid of his tumor at all risks, and M. Roux therefore operated in the following manner:—

He first made a long vertical incision from the os hyoides to the base of the sternum; this disclosed the tumor, which was readily dissected away, and but little blood was lost from the promptitude with which the vessels were ligatured. When, however, the recurrent nerve was divided, he had a paroxysm of dyspnœa, and the voice became extinct, and has since remained feeble. The operation, which lasted half an hour, was not followed by any bad symptoms.

The tumor extirpated, was irregularly ovoid, and enveloped in a cellulo-fibrous sheath. It was composed of numerous cysts of various sizes, some containiug a transparent serosity, others a cretaceous matter.

Another case was related by M. Hutin, senior-surgeon to the Invalids, and a third was communicated by M. Velpeau, in the name of Dr. Cabaret. Both were successful.

The discussion which originated in the detail of these cases showed distinctly that the dangers of the operation were considered very great. Velpeau, Roux, Begin, all expressed their opinion that the operation ought never to be undertaken, without admonishing the patient of the risk incurred.—*Prov. Med. and Surg. Journal, Dec. 11th* 1850.

